# Editorial: Contextualizing psychological assessment in Africa: COVID-19 and beyond

**DOI:** 10.3389/fpsyg.2023.1150387

**Published:** 2023-02-21

**Authors:** Seth Oppong, Richard Appiah, Given Hapunda, Jabulani Gilford Kheswa

**Affiliations:** ^1^Department of Psychology, University of Botswana, Gaborone, Botswana; ^2^Department of Psychology, University of the Witwatersrand, Johannesburg, South Africa; ^3^College of Health Sciences, University of Ghana, Accra, Ghana; ^4^Center for African Studies, Harvard University, Cambridge, MA, United States; ^5^Department of Psychology, University of Zambia, Lusaka, Zambia; ^6^Department of Psychology, University of Fort Hare, Alice, South Africa

**Keywords:** psychological assessment, psychological testing, test development, culturally appropriate assessment, African psychology, COVID-19

Historically, the science of Psychology and the practice of psychological assessment in Africa have been influenced by Western ideologies and practices (Nsamenang, 2007; Oppong et al., 2022). However, there are vast cultural differences among people of different social structures and value orientations. Therefore, there is a growing need for locally generated and validated tools to assess the strengths, capacities, and mental health of the African people (Oppong, 2017; Laher, 2019; Appiah et al., 2020; Oppong et al., 2022), who are often under-represented in global psychological research (Nielsen et al., 2017; Rad et al., 2018; Thalmayer et al., 2021). The overarching importance of generating and administering context-appropriate measures is that it strengthens the drive toward ensuring valid, reliable, and context-worthy assessment of mental health and psychological wellbeing among individuals, groups, and communities in the context of research and practice.

This Research Topic (RT), *Contextualizing Psychological Assessment in Africa: COVID-19 and beyond*, was aimed at curating evidence about the efforts by African researchers and practitioners at generating new tools, validating existing ones, and adapting the practices of assessment before, during, and after the COVID-19 pandemic through a collection of reports of original research, systematic reviews, and comprehensive narrative reviews.

To diversify the pool of potential contributors, we invited as many Africa-based and Africa-focused researchers and practitioners as possible through the use of: (1) the professional networks of the guest editors; (2) websites of African departments; (3) Africa-based and Africa-focused journals, books, and book chapters; and (4) invitations to individuals whose previous work appeared consistent with the goals of this RT. We invited a total of 269 prospective contributors, out of which 212 were unresponsive, 23 declined our invitation, 20 withdrew, and 14 confirmed their participation. We accepted 15 abstracts and expected additional 2 manuscripts from researchers who requested permission to submit after the deadline for receiving abstracts had elapsed. Of the 17 expected submissions, we received 12 full manuscripts. We accepted seven of these for publication. Unfortunately, the response to this RT was negatively affected by the Article Processing Cost (APC), given that many African universities do not have institutional agreements with *Frontiers* or do not support faculty with the APC. That researchers are often restricted by lack of funding to publish their work in high impact outlets like Frontiers is highly problematic and undermines our collective efforts in overcoming some of the limitations in hegemonic psychological theory, research, and praxis.

The seven (7) papers accepted for publication in this RT were mostly authored by South African and Ghanaian researchers. More recently, Serpell et al. (2022) reported a similar pattern of distribution of researchers who contributed to their RT (African Cultural Models in Psychology). Nonetheless, the samples for the studies published in this Research Topic were drawn from Ghana, Kenya, Mozambique, and South Africa, implying representation from West, East, and Southern Africa. The articles in this RT encompass a variety of topics, including a critical review (van der Merwe et al.), a perspective (van Vuuren et al.), development of ethical guidelines for online mental health screening tools (Hassem and Laher), an evaluation of an adaptation to a self-directed format of the Grade 9 Career Guidance Project (van Schalkwyk et al.), and application of approaches to construct validity (Anum; Khumalo et al.; Tadi et al.).

Together, the work by van der Merwe et al. and van Vuuren et al. provide guidance on how to approach the process of contextualizing assessment tools as well as the factors that influence the assessment process in the African context. For instance, van der Merwe et al identified contextual factors, the school learners' test-taking skills/familiarity and prior learning, and the school learners' cognitive abilities that affect the applicability of the test instrument when imported into other settings. Similarly, van Vuuren et al. raised the concerns of culture and language in the development of psychological tests, highlighting the challenges associated with test adaptation and translation and offered some guidance on how to navigate cultural and language issues in the development, adaptation, and translation of tools assessing intelligence and similar constructs.

Further, Hassem and Laher and van Schalkwyk et al. provided guidance on how to contextualize intervention programme or guidelines in the South African context. In this regard, Hassam and Laher presented guidance to researchers on the practical steps required to either contextualize an existing ethical guideline or develop new ones using a two-phase in-depth interview with mental health experts. On the other hand, van Schalkwyk et al. provided evidence via a mixed-methods design to evaluate teachers' support for the use of a self-directed manualised booklet to assist Grade 9 learners to explore their career interests and their knowledge of self. The reliance on teachers' evaluative feedback serves as key approach to assessing implementability of similar programmes. In particular, the assessment of relevance, appropriateness of methods of adaptation, and suggestions for improvements are important standards for evaluating any ethical guidelines.

The last set of studies (Anum; Khumalo et al.; Tadi et al.) sought to provide evidence of construct validity using different approaches. Tadi et al. employed the theory-consistent group differences approach (Gregory, 2021) to examine sex differences in the scores on Patient Health Questionnaire-9 (PHQ-9) and Generalized Anxiety Disorder-7 (GAD-7) in a sample of South African young adults using latent class analysis (LCA), inter-variable correlations, and group comparisons. Similarly, Khumalo et al. applied confirmatory factor analysis (CFA) and LCA to investigate the validity of the dual-continua model among young adults from Ghana, Kenya, Mozambique, and South Africa who completed the Mental Health Continuum—Short Form (MHC-SF) and PHQ-9. Both studies are exemplary as they utilized CFA (a traditional approach), LCA, large sample sizes, and cross-national samples to enhance the validity evidence they produced. Finally, Anum employed appropriate developmental changes and theory-consistent group differences approaches to investigate the relative impact of age and socio-economic status (SES) on fluid and crystallized intelligence. He showcased that the type of school attended (private or public) can reasonably be used as a proxy for SES among young children and offered practical steps on the use of appropriate developmental changes approach to construct validity.

We hope that this RT will inspire Africa-based researchers and practitioners to become more conscious of the need to adapt or generate context-responsive assessment tools, show preference for validated versions of tools, and contribute to culturally appropriate guidelines and testing practices in Africa and the Global South.

## Author contributions

SO developed the first draft. RA, GH, JK, and SO edited and improved it until a final version was agreed upon. All authors contributed to the article and approved the submitted version.
